# Bioeconomy imaginaries: A review of forest-related social science literature

**DOI:** 10.1007/s13280-020-01398-6

**Published:** 2020-10-09

**Authors:** Sara Holmgren, Dalia D’Amato, Alexandru Giurca

**Affiliations:** 1 Department of Urban and Rural Development, Swedish University of Agricultural Sciences, Ulls väg 27, 750 07 Uppsala, Sweden; 2 Helsinki Institute of Sustainability Science, Department of Forest Sciences, Faculty of Agriculture and Forestry, University of Helsinki, Latokartanonkaari 7, P.O. Box 27, 00014 Helsinki, Finland; 3 Chair of Forest and Environmental Policy, Faculty of Environment and Natural Resources, University of Freiburg, Tennenbacherstr. 4, 79106 Freiburg, Germany

**Keywords:** Circular bioeconomy, Equity, Forest-based bioeconomy, Knowledge-based economy, Sustainable transformation, Sustainability transitions

## Abstract

This review article examines how social science literature co-produces various imaginaries of forest-based bioeconomy transformations and pathways for reaching desired ends. Based on an analysis of 59 research articles, we find that despite a growing number of social sciences studies on the forest-based bioeconomy, much of the research tends to replicate a bioeconomy imaginary articulated in EU and national bioeconomy policies and strategies. Accordingly, the research primarily reproduces a weak approach to sustainability, which prioritize economic growth and competitiveness. Expectations are largely directed at national and regional corporate interests and forest industrial renewal, while the state has a supportive rather than restricting role. We discuss the findings against the role of social sciences, and conclude that social science scholars may adopt various strategies if interested in opening up forest-based policy debates and offer alternative imaginaries of sustainable bioeconomy transformations.

**Electronic supplementary material:**

The online version of this article (10.1007/s13280-020-01398-6) contains supplementary material, which is available to authorized users.

## Introduction

Global societies need to fundamentally restructure production and consumption systems to tackle climate change, resource depletion, and widening social inequality. This has led to a substantial focus on sustainability transformations in both science and politics (Abson et al. 2017; Hölscher et al. 2018). Considering that transformations are almost always imbued with change towards greater sustainability, they are inevitably normative endeavours that explicitly (or not) carry with them ideas of desired futures. This may include changes needed for realizing desired futures, the instruments that should be used to implement such changes, and the agents driving change (Yusoff and Gabrys 2011; Wangel 2011; Jasanoff 2015). Simultaneously, value judgments influence how societies value the well-being of current generations compared to that of future generations, whether intrinsic or instrumental values are attributed to ecological systems, whose interests and preferences are heard or marginalized, and how the risks and costs associated with sustainability transformations are socially and economically distributed (Pickering and Persson 2019).

Based on an understanding of science and policy as interlinked processes of collective meaning making, we argue that researchers have an important say in how sustainability transformations are envisioned and translated into practice. In this review article, we therefore examine how researchers co-produce certain visions or ‘imaginaries’ of sustainable futures as more desirable than others (Wesselink et al. 2013), thereby naturalizing or challenging various sustainable development trajectories. Here, the concept of ‘imaginary’ refers to collective ways of thinking and seeing, and it is understood as an inherent part of scientific knowledge production, which may configure and/or disrupt present political decision-making along with behaviours (Stirling 2008; Yusoff and Gabrys 2011; Wesselink et al. 2013).

Empirically, the review focuses on research engaging in the forest-bioeconomy, i.e. a particular transformation process that has gained momentum worldwide over the past decade (Staffas et al. 2013). The bioeconomy is an elaborate concept, interpreted differently by scholars, policymakers, and practitioners. In general, it is concerned with unlocking and commercializing the potential of biological resources and their functions through knowledge and innovation. Existing literature reviews suggest that three visions may be identified with different foci: biomass resources, biotechnology, and agroecology. While the first two are well represented in political strategies, the third one emerges from scientific literature (Bugge et al. 2016; Hausknost et al. 2017; Meyer 2017; D’Amato et al. 2019). Critical voices in the scholarly literature have already pointed out how both biomass- and biotechnology-oriented bioeconomy visions may be compared to a new mode of capital accumulation (Birch and Tyfield 2013; Goven and Pavone 2015).

In policy making and research, the bioeconomy is generally associated with the idea of replacing fossil-based resources with bio-based ones by means of knowledge development and innovation. It thus holds an optimistic promise of coupled decarbonization, sustainability, and green growth (Ahlqvist and Sirviö 2019). Accordingly, the Organization for Economic Cooperation and Development (OECD), the European Union (EU) along with a number of nations worldwide have adopted bioeconomy strategies (Dietz et al. 2018).

Considering the broad scope of bioeconomy research, we have limited our review to social-scientific publications that explicitly deal with the forest-based sector. This is motivated by the forest-based sector being promoted as one of the main pillars of the European bioeconomy strategy (European Commission 2018). Forests are important sources of renewable biomass in many countries and are central to the maintenance of ecological processes of fundamental relevance for human well-being (e.g. climate mitigation and adaptation, water and soil protection, biodiversity conservation, recreation and cultural ecosystem services). Furthermore, funding for research related to the forest-based bioeconomy is increasing in Europe (Lovrić et al. 2020). Bioeconomy imaginaries in the scholarly research are likely to have major implications for how these forest values are balanced and prioritized in policymaking and whose perspectives and interests concerning forests are heard, or whose are marginalized.

Despite increasing funding for research related to the forest-based bioeconomy, social sciences remain largely underrepresented in the scientific disciplines dominating bioeconomy projects, which primarily are associated with the natural sciences, engineering, chemistry, or other technical disciplines (Giurca and Metz 2018; Korhonen et al. 2018a, b; Toppinen et al. 2019a, b). However, the bioeconomy is more than simply or primarily a techno-scientific or economic endeavour, as the relevance of social science is increasingly acknowledged (Goven and Pavone 2015; Sanz-Hernández et al. 2019). Social sciences are fundamental for gaining a deeper understanding on how policies, market forces, actors, and knowledge claims interact and shape conditions for the bioeconomy (Kleinschmit et al. 2014). While bibliometric literature reviews of the social science literature exist (Sanz-Hernández et al. 2019; Paletto et al. 2020), no study in the scientific literature has so far examined how social science research, through its knowledge making, co-shapes the meaning and governance of the forest-based bioeconomy. By doing so, our study adds additional insights to the expanding social science scholarship focusing on sustainability transformations in general and on the forest-based bioeconomy transformation in particular.

## Analytical framework

### Conceptualizing sustainability transformations

Through the launch of the Brundtland report in the late 1980s, sustainable development became a widespread concept originally associated with a process of change in which the exploitation of resources, investments, orientation of technological development, and institutional change are made consistent with future and present needs. The report further concluded that this process rests on political will and that it requires ‘painful’ choices (WCED 1987, p. 16). Since the launch of the Brundtland report, ‘transformation’ has become a buzzword in political and scientific discourses, capturing the process of change called for in the report. More recently, ‘transformation’ is used in the context of the sustainable development goals, i.e. in the quest of enhancing their ‘transformative potential’ (Hajer et al. 2015). A wide range of research has emerged attempting to understand, analyse, and support sustainable transformations, which generally refer to radical, non-linear, and structural changes in social, technological, institutional, and economic systems that aim for various degrees of fundamental shifts in human–environmental interactions (Hölscher et al. 2018). Still, little consensus remains concerning the features that actually make changes in human–environment systems ‘transformational’ (Feola 2015).

In this article, we use the term transformation as a representation of fundamental changes in, e.g. forestry, energy markets, identities, livelihoods, ethics, and governance (c.f. Feola 2015, p. 5) that aim for shifts in human–environmental relations. We approach sustainability transformations as complex challenges characterized by uncertainties, contestations, and urgency, which are often confronted by cultural, social, and political barriers to change (Miller and Wyborn 2018). Accordingly, sustainability transformations are long-term democratic projects where definitions of new socially shared meanings, collective behaviours, and the inclusion of new actors are central to any practical attempts to link place-based and global approaches, local community interests with traditional institutional actors, and short- and long-term priorities. Transformational actions thus require more than developing the right technologies, institutions, markets, and metrics (Mancebo and Sachs 2015), which are often the focus of scientific knowledge produced in bioeconomy-related projects (Giurca and Metz 2018; Korhonen et al. 2018a, b; Lovrić et al. 2020).

While still emerging, social science research on the bioeconomy has provided important insights regarding the ambiguity of the bioeconomy concept and called for more inclusive and broader bioeconomy-related debates. Some scholars describe the bioeconomy as a ‘political project’ that is meant to bring about a particular set of political–institutional changes that will shape possible future actions (Goven and Pavone 2015). Others define it as a ‘mixed-source discourse’, where classical forest and environmental discourses are reframed in the bioeconomy context, while others (e.g. limits to growth) are neglected (Pülzl et al. 2014). Several studies have addressed the relationship between the bioeconomy and sustainability concepts. For example, in an analysis of the EU bioeconomy policy framework, Ramcilovik-Suominen and Pülzl (2016) conclude that the narrow focus on economic growth will not help tackle the sustainability challenges that societies currently face. Pfau et al. (2014) further observe that sustainability concerns are weakly defined and poorly specified in bioeconomy-related research. In general, the bioeconomy concept is characterized by an anthropocentric approach and weak sustainability,^1^ which imply that economic growth is seen as a prerequisite for solving environmental problems (Loiseau et al. 2016; Liobikiene et al. 2019).

More critical and interpretive analyses argued that additional attention needs to be directed at the politics of the bioeconomy to avoid unintentional legitimatization of mainstream bioeconomy visions (Goven and Pavone 2015; Ahlqvist and Sirviö 2019). From this perspective, the role of social sciences goes beyond offering a deeper understanding, e.g. of how the bioeconomy is shaped or providing recommendations for more efficient or democratic implementation of desired goals articulated in various bioeconomy strategies. The task is rather to analyse the norms, values, and power relations that implicitly or explicitly underpin and shape bioeconomy-related projects, which may have major effects on social and environmental justice and equity and ultimately on the transformative potential of the bioeconomy (Ahlqvist and Sirviö 2019). Such analysis must also include research practices, which imply that we need to reflect upon and be more open about the values that underpin our research and how they shape the way we advocate for social, cultural, and political change (Andersson and Westholm 2019; Pickering and Persson 2019; Wyborn et al. 2019). By reflecting on how we as social scientists co-produce different imaginaries of the forest-based bioeconomy transformation, our study adds to this critical strand of literature on the bioeconomy and sustainability transformations.

### ‘Imaginaries’

‘Imaginaries’ are here understood as ways of seeing and thinking that creates the conditions for material interventions in the world (Yusoff and Gabrys 2011). The term ‘imaginaries’ has become increasingly established in the interpretive analysis of social and political phenomena (Yusoff and Gabrys 2011; Jasanoff 2015). Jasanoff (2015, p. 4) broadly defines the term as ‘collectively held, institutionally stabilized, and publicly performed visions of desirable futures, animated by shared understandings of forms of social life and social order attainable through, and supportive of advances in science and technology’.

Theoretically, the imaginary concept is located within the co-productionist framework and has been theorized in interpretive policy studies (Wesselink et al. 2013), in Critical Discourse Analysis (CDA) (Levidow and Papaioannou 2013), and future studies (Wangel 2011). According to the co-productionist approach, science, technology and social order are, at all times, the result of a co-production process, which generates new imaginaries, technologies and norms, policy guidelines and power relationships (Jasanoff 2005; Jasanoff and Kim 2009). Imaginaries are not tied to future possibilities solely through scientific or technological practices (Jasanoff and Kim 2009; Goven and Pavone 2015), and are not to be equated with policy agendas as they are less instrumental, less explicit and goal-oriented. Still they can be associated with active exercises of state power, i.e. through the allocation of funds for development priorities, the investment in certain infrastructures or technologies and through political cooperation or opposition (Jasanoff and Kim 2009).

Empirically, ‘imaginaries’ have been particularly employed in studies of energy transitions helping scholars understand how various state visions of the future shape the actions, behaviours, and political interventions in present energy systems (Jasanoff and Kim 2009; Levidow and Papaioannou 2013; Kuchler 2014; Cherry et al. 2017). The concept has more recently also been applied in bioeconomy-related research (Pavone and Goven 2015, 2017). Here, we expand the use of the concept to the area of forest-based bioeconomy research to illustrate how we as social scientists—an important and heterogeneous group of social actors with particular authority in climate-, forest-, environmental-policy and sustainability governance—collectively make sense of sustainable development, of forests and human-forest relations, and construct and/or resist different visions of bioeconomy transformations.

To answer this broad question, we conduct an interpretative analysis of social science articles related to forest-based bioeconomy transformations. Drawing on the framework developed by Wangel (2011, p. 875) we operationalise our analysis through the means of three interrelated questions:

1. To what ends are forest-based bioeconomy transformations considered desirable?
2. What changes are called for in order to pursue desired forest-based bioeconomy transformations?
3. Which measures and agents of change are deemed relevant for forest-based bioeconomy transformations?

Originally developed to investigate how social structures and agency have been included in back-casting studies for sustainable development, these questions have structured our empirical analysis, which is elaborated in the Methods Sect. 3.2.

## Methods

### Data collection

Scientific publications are an important output in any given research field and offer an important starting point for exploring scientific imaginary practices. To identify relevant scientific articles for this review, we performed a search in Web of Science and Scopus restricted to specific disciplines in the realm of social sciences across all years. Considering that forest-based bioeconomy research is multidisciplinary, these two databases were selected because they include research across a wide range of scientific fields.

Social sciences encompass many branches and include, but are not limited to, cultural (or social) anthropology, sociology, social psychology, political science, and business and economics. Social and economic geography and certain areas of education are also often included (Nisbet 2019).

Given the different definitions and overlaps of these branches and their prominence in various issue areas, such as bioeconomy transformations, we limited the scope of our analysis to five branches: policy research, economics, business administration, innovation studies, and society and technology studies. These branches have previously been observed as particularly relevant for a broadened understanding of forest-based bioeconomy transformations (Kleinschmit et al. 2014). These branches include both positivist and interpretivist philosophies. Positivist social scientists may use methods resembling those of the natural sciences such as life-cycle analysis of forest-based bioeconomy products and material flows. Interpretivist social scientists on the other hand may use social critique or try to deconstruct policy narratives or forest-based bioeconomy discourses. Our selection of social-scientific branches allows for the inclusion of a broad spectrum of different ontologies and epistemologies co-producing different forest-based bioeconomy imaginaries.

**Fig. 1:**
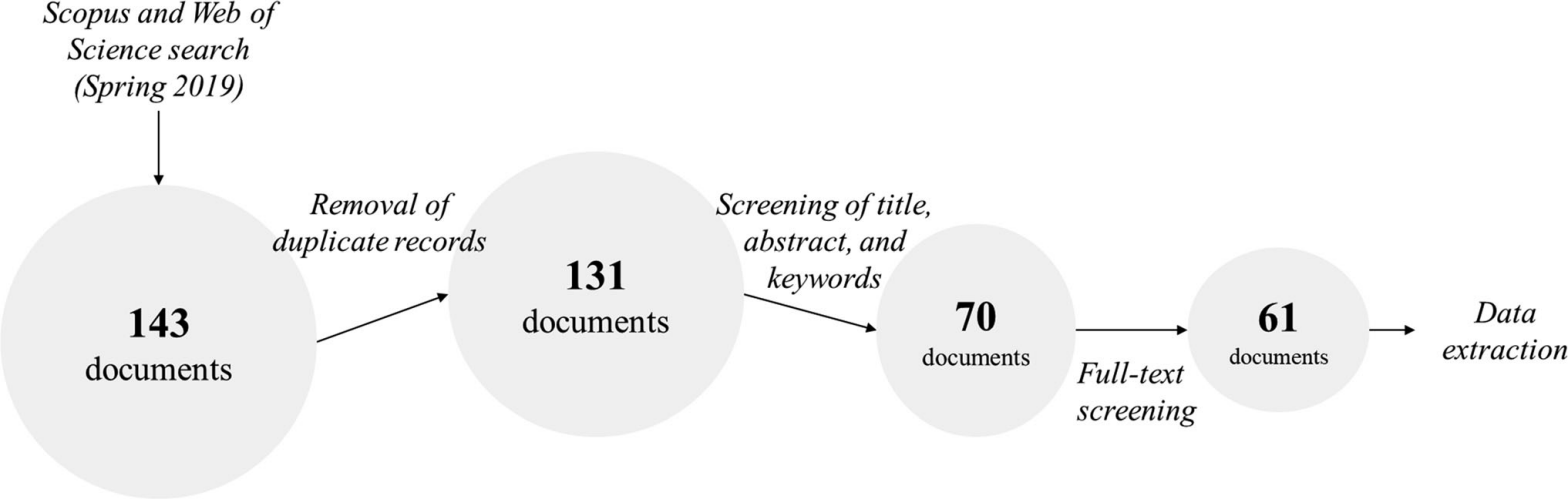
Process of data collection

The search was restricted to articles published in English and containing keywords related to the bioeconomy and forests (Table 1). In Scopus, we searched for scientific articles (excluding, e.g. conference proceedings and books) using the ‘abstract and keywords’ function (TITLE-ABS-KEY). We coupled this search with a search in Web of Science, where we performed a topic search (TS) of review articles, which in total resulted in 143 documents (Fig. 1).

**Table 1:** Strings used for the literature search in Scopus and Web of Science

Database	Search string	Records found
Scopus	TITLE-ABS-KEY ( "bioeconomy" OR "bio economy" OR "bio-economy" OR "biobased economy" OR "bio-based economy" OR "bio based economy" AND "wood*" OR "forest*") AND ( LIMIT-TO ( SUBJAREA, "SOCI") OR LIMIT-TO ( SUBJAREA, "BUSI") OR LIMIT-TO ( SUBJAREA, "ECON") OR LIMIT-TO ( SUBJAREA, "DECI") OR LIMIT-TO ( SUBJAREA, "MULT") OR LIMIT-TO ( SUBJAREA, "ARTS")) AND ( LIMIT-TO ( DOCTYPE, "ar") OR LIMIT-TO ( DOCTYPE, "re") OR LIMIT-TO ( DOCTYPE, "ip"))	104
Web of Science	(TS = ("bioeconomy" OR "bio economy" OR "bio-economy" OR "biobased economy" OR "bio-based economy" OR "bio based economy") AND TS = ("wood*" OR "forest*")) AND LANGUAGE: (English) AND DOCUMENT TYPES: (Review) Refined by: WEB OF SCIENCE CATEGORIES: (FORESTRY OR ENVIRONMENTAL STUDIES OR ENVIRONMENTAL SCIENCES OR ECONOMICS OR REGIONAL URBAN PLANNING OR ENERGY FUELS) Timespan: All years. Indexes: SCI-EXPANDED, SSCI, A&HCI, CPCI-S, CPCI-SSH, BKCI-S, BKCI-SSH, ESCI, CCR-EXPANDED, IC	39

After eliminating double records, the search resulted in 131 articles. In a second step, we manually screened the abstracts, titles, and keywords aided by the software Abstrackr (Wallace et al. 2012) using three criteria to accept or reject the remaining articles: (1) the word ‘bioeconomy’ or synonyms had to be mentioned^2^; (2) forests, the forest sector, or wood had to be mentioned; (3) social science had to be the realm of investigation. Two co-authors performed the screening independently and disagreements were resolved through discussion. This manual screening resulted in 70 articles.

### Analytic procedure

For the interpretive analysis, the full texts of the 70 articles selected for the review were equally distributed among the three authors. In this phase, additional articles were excluded, as they proved to not deal with the forest-based bioeconomy beyond the abstract, title, or keywords, which resulted in 59 articles in total (see Appendix S1). To offer an overview of where and by whom the research is produced, we summarized descriptive statistics of the journals in which the articles were published, the gender and country affiliation of the first authors, and the geographical scope of the studies. For the interpretive analysis, each author was responsible for a subset of the reviewed articles that did not include any of our own publications. We read through the articles and coded the texts manually in accordance to the questions described in “Conceptualizing sustainability transformations” section. As the three questions overlap and imply some repetition, listing them separately simply served as a heuristic. To summarize our findings, we identified themes abductively and constructed subcategories in relation to each question. The subcategories are not mutually exclusive, which imply that a publication may fit more than one subcategory.

Based on the summaries of the individual findings, the first author merged and labelled identified subcategories and elaborated on the analysis. After that, the co-authors complemented and further developed the analysis to make sure it reflected the analysed literature. As the review focuses on broader patterns of thinking and interpreting the forest-based bioeconomy transformations, the analytical section does not present each individual article reviewed. To provide insight into how we have interpreted and categorized the material, we provide references to articles that are illustrative for the identified imaginaries.

## Analysis

### Overview of the literature

The reviewed articles were published in a variety of international journals, as follows: Journal of Cleaner Production (JCP): 15 papers; Forest Policy and Economics (FPE): 10 papers; Sustainability (Sus): 7 papers; Scandinavian Journal of Forest Research (SJFR): 4 papers; Ecological Economics (EE): 4 papers; Technology and Society (TS): 2 papers; and the Canadian Journal of Forest Research (CJFR): 2 papers. The remaining 19 articles were published in journals ranging from various economics-focused journals to more natural science-oriented journals in the area of forest management, agriculture, and ecology. Others were published in journals specialized in business and management.

**Fig. 2:**
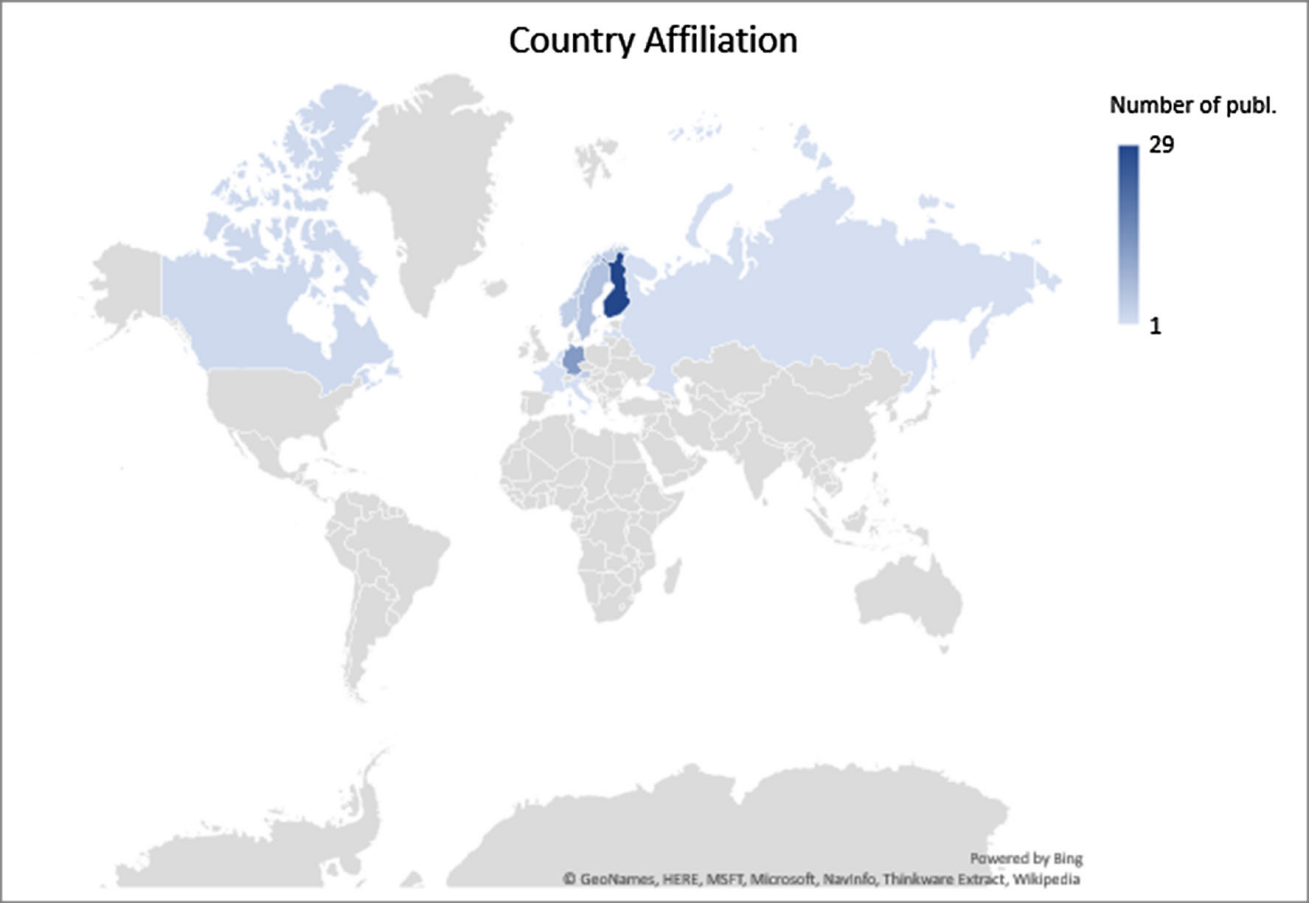
Affiliation of the first authors of the reviewed articles

As illustrated in Fig. 2, the majority of first authors (29 papers) are affiliated with Finnish-based research institutions; followed by German-based institutions (13 papers); Swedish-based institutions (6 publications); other Northern European institutions such as Norway (4 publications); and Central-Western European institutions, e.g. Austria (4 publications), Italy, the Netherlands, France, Belgium, and Latvia (one publication each). Canada and Russia have one affiliation per country.

**Fig. 3:**
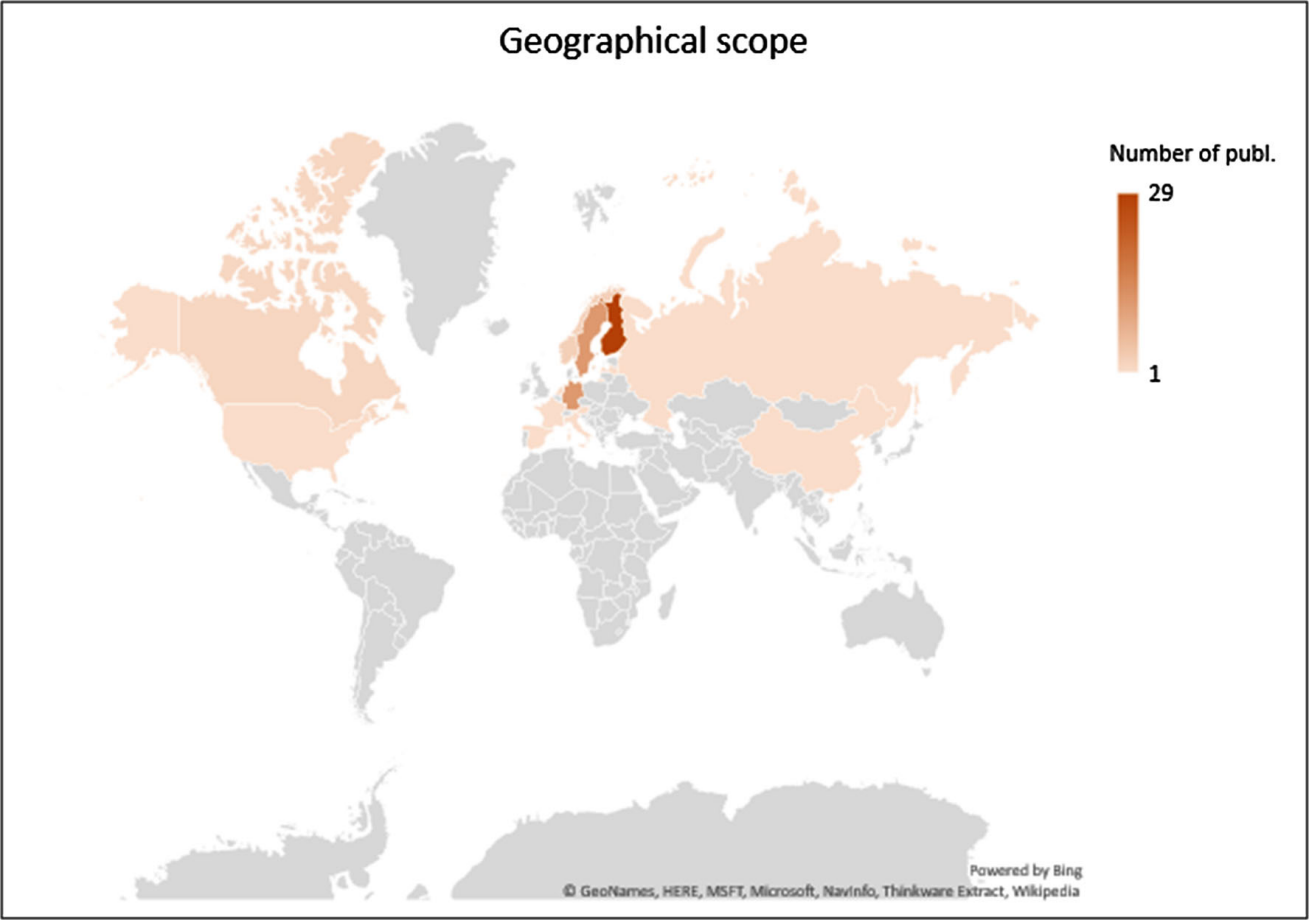
Geographical area studied in the reviewed articles

The geographical focus of the articles is usually the same as the country affiliation of the authors (Fig. 2). However, certain articles take a European Union (7 papers) or global perspective (4 papers). Similar to country affiliation, the geographical scope of the articles is dominated by Finland (29 papers); followed by Germany and Sweden (12 papers each). The rest of the articles focus on other European countries such as Norway (3 papers), Italy, The Netherlands, Spain, Latvia, Austria, France, and Russia (one paper each). Publications focusing on countries and regions outside the European continent include Canada, USA, and Hong Kong (Fig. 3). Out of the 59 publications considered in this analysis, 30 were first-authored by male researchers and 29 by female researchers.

### To what ends are forest-based bioeconomy transformations considered desirable?

In this subsection, we elaborate on the rationales underpinning forest-based bioeconomy transformations, i.e. to what ends are forest-based bioeconomy transformations considered desirable? We identified three types of rationales recurring in the reviewed articles. We describe them in Table 2 and in “The forest-based bioeconomy as a means to decarbonize and maintain economic growth”–“The forest-based bioeconomy as fundamental societal transformation” sections. Note that the rationales increasingly problematize the forest-based bioeconomy, ranging from affirmative (The bioeconomy as a way to decarbonize and maintain economic growth) to descriptive normative (The bioeconomy as a potential pathway towards sustainability) to critical in a theoretical sense (The bioeconomy as a fundamental societal transformation).

**Table 2:** Types of rationales underpinning forest-based bioeconomy transformations

Forest-based imaginaries	Description of rationale	Examples of reviewed documents aligning with each type
The forest-based bioeconomy as a way to decarbonize and maintain economic growth	- Replacement of fossil-based materials and fuels with bio-based ones - Assumption of sustainability a priori - Focus on economic growth, efficient production, and use of forest biomass - Replication of rationales articulated in bioeconomy policies, primarily the EU strategy	Blair et al. (2017), Giurca and Späth (2017), Hagman et al. (2018), Hildebrandt (2019), Hurmekoski et al. (2018), Husgafvel et al. (2018), Lehtonen and Okkonen (2013), Lilja and Moen (2017), May et al. (2017), Pelse et al. (2018) Sikkema et al. (2017), etc
The forest-based bioeconomy as a potential pathway towards sustainability	- Questioning of sustainability assumptions - Advocating a more balanced sustainable bioeconomy agenda, including social and environmental goals - Problematizing ambiguity and context dependence of the bioeconomy concept from democratic and/or efficiency perspectives	Giurca and Metz (2018), Hagemann et al. (2016), Jarre et al. (2020), Johansson (2018), Kleinschmit et al. (2017), Näyhä (2019), Siebert et al. (2018), Takala et al. (2019) etc
The forest-based bioeconomy as a fundamental societal transformation	- A priori adoption of a critical approach to dominating forest-based bioeconomy policy visions and their potential to deliver sustainable transformation - Explicit elaboration of sustainable and desirable ends of forest-based bioeconomy transformations, with ecological sustainability as the main premise - Arguments for a just, equal, and democratic transformation of society as a whole, where natural resources are valorised for local benefits and power asymmetries (North–South) are addressed	Ahlqvist and Sirviö (2019), Grundel and Dahlström (2016), Kröger (2016), Kröger and Raitio (2016), Mustalahti (2018) etc

#### The forest-based bioeconomy as a means to decarbonize and maintain economic growth

A dominant way of representing the desired ends of forest-based bioeconomy transformations is to replicate the rationales articulated in existing national, regional, or international bioeconomy strategies, most notably the EU Bioeconomy strategy from 2012 (European Commission 2012). Accordingly, bioeconomy transformations are seen as a means to achieve more efficient forest resource production/use in the present to allow for decarbonization and maintained economic growth (Hagman et al. 2018; Husgafvel et al. 2018; Pelse et al. 2018), which is also in line with the EU bioeconomy policy framework. Studies imagining the forest-based bioeconomy as a means to decarbonize and maintain economic growth tend to not articulate or discuss the normative underpinnings of the conducted research or the desired outcomes of the bioeconomy. In general, the studies do not include any explicit comments on whether the authors agree or disagree with the policy formulations and they do not elaborate on alternatives. Abstaining from commenting on rationales articulated in bioeconomy policies does not necessarily mean that the authors fully agree with the political project as it is formulated or share its normative underpinnings. Still, the mere replication inevitably suggests a naturalization of the political rationales articulated by the EU strategy.

However, replicating the 2012 EU Bioeconomy policy framework as the main rationale behind the analysis inevitably leads to a stronger focus on the economic dimension of sustainability. Empirically, this implies focusing on issues connected to industrial forestry, such as forest management, industrial supply streams optimization, strengthening industrial cooperation partners (Hildebrandt et al. 2019), rejuvenating communities and industrial sectors, and ensuring growth and competitiveness of certain products and services with the help of technology (Lehtonen and Okkonen 2013; Blair et al. 2017). Moreover, certain articles represent the bioeconomy as already existing but needing to expand and become more efficient at utilizing forest-based resources (Lilja and Moen 2017; Myking et al. 2017).

The articles replicating bioeconomy policies tend to represent forest-based bioeconomy transformations in a narrow, production-oriented sense without addressing more fundamental cultural, social, political, and economic challenges. Little consideration is given to limits to production/consumption or to the social and environmental implications of an expanded forest-based bioeconomy transformation.

#### The forest-based bioeconomy as a potential pathway towards sustainability

Studies seeing the forest-based bioeconomy as a potential pathway towards sustainability, approach the concept in a broader sense and do not assume that bioeconomy transformations are sustainable per se. Sustainability is typically referred to as the balancing of social, ecological, or economic dimensions, and/or in terms of sustainable development goals (D'Amato et al. 2019; Hurmekoski et al. 2019; Näyhä 2019; Takala et al. 2019). This argumentation generally problematizes the sustainability claims made in bioeconomy policies, and the authors typically acknowledge the ambiguity of the bioeconomy and/or the sustainability concepts, while paying analytical attention to the diversity of understandings and visions of the bioeconomy in various contexts (Pülzl et al. 2014; Kleinschmit et al. 2017; Takala et al. 2019). Identifying diverse interpretations of the forest-based bioeconomy is often part of the empirical enquiry, and the way the bioeconomy is conceptualized and realized is problematized from various perspectives, e.g. democratic (the diversity of actors involved, legitimacy) (Johansson 2018), efficiency (policy integration, implementation, economic) (Hagemann et al. 2016), and/or its implication on social and environmental sustainability (Kleinschmit et al. 2017; Johansson 2018; Mustalahti 2018). Although these studies do not accept the bioeconomy as it is, they generally acknowledge the potentially good merits of the forest-based bioeconomy as part of the solutions to sustainability challenges. However, most studies in this category point to the many improvements that remain to be made regarding the way the forest-based bioeconomy is defined and implemented. Despite the problematizing approach and the will to improve, few studies explicitly reflect on or articulate the normative underpinnings of the research conducted or the value judgments shaping the recommended improvements.

#### The forest-based bioeconomy as fundamental societal transformation

A less common way of interpreting the bioeconomy is as fundamental societal transformation. These studies typically adopt and articulate a priori critical approach to dominant forest-based bioeconomy conceptions and their potential to deliver sustainability. To various degrees, they explicitly elaborate on what is seen as sustainable and/or desirable outcomes of bioeconomy transformations and address various aspects of power. Such discussions often entail calls for just, equal, and democratic transformation of society as a whole (at all levels), where social, ecological, and economic dimensions are balanced (Grundel and Dahlström 2016), natural resources are valorised for local benefits (particularly in rural areas) (Ahlqvist and Sirviö 2019), and power asymmetries in the global political economy are addressed (Kröger 2016).

Rather than focusing on forest industrial perspectives, global competitiveness, and technological innovation, these studies emphasize the importance of local and regional actors in bioeconomy-related governance, social and economic justice, and environmental requirements/limits, and they additionally emphasize ecological sustainability as a primary premise (Kröger and Raitio 2016; Mustalahti 2018). In comparison to the imaginaries of desired ends, the studies representing the bioeconomy as a fundamental societal transformation advocate more radical and fundamental changes in the way we live and organize societies beyond the capitalist, growth-oriented economy.

### What changes are called for in order to pursue desired forest-based bioeconomy transformations?

While the reviewed articles tend to avoid engaging in and/or discussing in detail the reasons why bioeconomy transformations are desirable and what constitutes a sustainable bioeconomy, suggestions and recommendations on what is to be changed tend to be more explicitly articulated. Such problem-solving information, however, implicitly indicates the articles’ stand points regarding why bioeconomy transformations are desirable. Changes that are deemed relevant in the reviewed documents may be grouped into three types. We synthetized these in Table 3 and Sects. 4.3.1–4.3.2. Change types occur at the industry level (Industrial renewal/mutation), at the land-use level (Forest management practices), or at a broader scale (Systemic change at social, political, and/or economic level).

**Table 3:** Types of changes needed to pursue desired forest-based bioeconomy transformations

Types of change needed for forest-based bioeconomy transformations	Description	Examples of reviewed documents aligning with each type
Industrial renewal/mutation	- Changes supporting industrial renewal and expansion - Forest industry production patterns as the key objects of change to support the expansion of the bioeconomy - Changes in infrastructures, technologies, and materials supporting bio-based products as replacements of fossil-based ones, while maintaining economic growth	Bennich et al. (2018), Giurca and Späth (2017), Hildebrandt et al. (2019), Hurmekoski et al. (2018), Jernström et al. (2017), Korhonen et al. (2018a, b), Näyhä (2019), Toppinen et al.(2018; 2019a, b) etc
Forest management practices	- Considerations and suggestions concerning forest management practices to mitigate trade-offs between ecosystem services or between sustainability dimensions/policy goals - Promotion of a more diversified forest management to balance forest values - Intensified wood production remains a means to substitute fossils and uphold economic growth	Bennich et al. (2018), Eyvindson et al. (2018), Matthies et al. (2018), Myking et al (2017), Sikkema et al. (2017) etc
Systemic change at social, political and/or economic level	- Advocating more or less radical changes at the system level - Suggestions for new forms of valuing nature and new forms of consumer behaviours (de-growth, reduced consumption), including waste reduction through circular and sharing societies - Advocating for more emphasis of environmental and social concerns in governing the bioeconomy (environmental impacts/ecological limits, equity and justice, local perspectives)	Ahlqvist and Sirviö (2019), Grundel and Dahlström (2016), Jarre et al. (2020), Kleinschmit et al. (2017), Kröger (2016) etc

#### Industrial renewal/industrial mutation

A common object of change is associated with forest industries and the process of industrial renewal/industrial mutation, which is intimately related to the desire to end the path-dependency on fossil fuels (Pannicke et al. 2015) while maintaining the competitiveness and/or growth of forest and/or wood-based industries. Industrial renewal and mutation require changes in social and technological structures. However, rather than linking fossil fuel dependence to the forest sector and elaborating on how forest industries may decarbonize (e.g. their production processes and transports and thereby reduce their climate impact), the focus of these studies is often directed at strengthening the marketability of wood-based products in relation to fossil-based ones, whereas social and environmental implications of the substitution are little addressed.

Studies focusing on industrial renewal generally consider the substitution of fossil resources with forest-based resources (especially wood and forest-based residues) as the basic point of departure. As a consequence, change is primarily initiated through technical processes meant to optimize and increase production in the forest-based industry. Biomass is to be used more efficiently through various processes (e.g. cascade use, increased circularity, by increasing the yield of recycled fibre, life-cycle thinking) and technologies (most prominently biorefineries) to obtain a range of bio-based products and chemicals (Lilja and Moen 2017; Hagman et al. 2018; Husgafvel et al. 2018; Temmes and Peck 2020). The forest-based bioeconomy transformation is thus imbued with industrial renewal and innovation involving new technologies, materials, production processes, and infrastructures (Korhonen et al. 2018a, b) along with increased use of wood in the construction sector to store carbon in products with longer life spans and to substitute fossil-intense materials such as steel and concrete (Toppinen et al. 2018; Toppinen et al. 2019a, b; Lazarevic et al. 2020).

In addition to technological and physical objects of change, certain articles also include social structures in the sense that they discuss or examine the forest industries approach/attitude to resource utilization and consumer preferences (Bennich et al. 2018; Näyhä 2019). Some argue that forest-based industries and their expected/predicted process of modernization are dependent on continuous efforts to cooperate (‘industrial symbiosis’) with other sectors (e.g. the agricultural sector) and industries (most prominently the chemical industry) (Hildebrandt et al. 2019). This entails building new social relationships and developing new patterns of social interaction beyond established networks, including more diverse societal actors such as nongovernmental organizations (NGOs), policymakers, and citizens (Giurca and Metz 2018; Korhonen et al. 2018a, b; Giurca 2020). Other studies focus on consumer preferences and suggest that industries that do not adjust to consumers’ increased environmental standards will soon be outdated (Pätäri et al. 2017).

#### Change in forest management practices

Forest management practices are another dominant object of change that involve material and social dimensions. Here the focus is on production, management, and extraction of forest biomass rather than the products and their utilization. Certain studies advocate intensified wood production as a means to reduce fossil dependence and simultaneously uphold economic growth and typically replicate the goals and desired ends articulated in bioeconomy policies (Myking et al. 2017). Other studies also advocate change in forest management practices that have a more problematizing or critical approach to the sustainability of dominant bioeconomy representations. These studies warn that land use aimed at maximizing timber production is likely to entail trade-offs with other ecological or social goals (Eyvindson et al. 2018). Scholars in this latter category typically argue for a shift towards diversified forest management and (ecosystem) services, e.g. improved forest management or protection of carbon-rich forests (Sikkema et al. 2017), a change in norms and practices among the actors involved in forest management and among the industries currently adjusted to intensive biomass production. In general, these studies do not take the sustainability of the bioeconomy for granted but consider it to be conditioned by socio-ecological factors (Bennich et al. 2018).

#### Systemic change (in the social, political, and/or economic system)

Compared to the literature focusing on industrial renewal/mutation or forest management practices, another and less common thread envisions change in broader and more systemic terms. Some argue that more emphasis is needed on environmental concerns in forest-based bioeconomy decision-making (Kleinschmit et al. 2017) and/or that the social dimension must be integrated in bioeconomy policymaking to be sustainable and/or efficient, just, and equal (Grundel and Dahlström 2016; Cavicchi et al. 2017; Borgström 2018; Mustalahti 2018; Ahlqvist and Sirviö 2019). Others argue that there is need for new ways of conceptualizing sustainability in relation to the forest-based bioeconomy transition, which requires new ways of thinking and acting (Takala et al. 2019). Certain articles call for fundamental changes in societal norms and values, which involve radical altering of the economic system including new forms of valorising nature, redistributing/reallocating wealth between centres and peripheries (Ahlqvist and Sirviö 2019), and overcoming global North–South power asymmetries (Kröger 2016). Others focus away from linear thinking towards circularity, which entails changes in production processes involving a move from through-put to circularity along with changed consumer behaviours to drastically reduce waste generation (Jarre et al. 2020). Rather than emphasizing technological innovation, Grundel and Dahlström (2016) stress the importance of social innovation, including new social practices that contribute to regional forest-based bioeconomy development.

### Which measures and agents of change are deemed relevant for forest-based bioeconomy transformations?

In this subsection, we elaborate on how forest-based bioeconomy transformations are imagined to occur, i.e. what measures are needed. This is deeply interconnected to the agents expected to drive change. Types of measures and related actors identified in the reviewed documents are described in Table 4 and  “Transformation through political support”–“Transformation through collaboration, inclusion, and transparency” sections. The three measure types range from more top-down mixes (i.e. transformation through political support/restrictions) to more diffuse approaches (i.e. transformation through inclusion, collaboration, and transparency; transformation through information). In the first type of measure, agents of change are represented by a triple helix, including policy, academia, and industries. The second type calls for more inclusive participation of a broader range of actors, including smaller players from various societal realms (entrepreneurs, farmers/forest owners, environmental NGOs (ENGOs), and citizens). The third type is concerned with public and private actors engaging with voluntary monitoring/information related to forest-based bioeconomy goals and impacts.

**Table 4:** Types of measures and related agents of change for forest-based bioeconomy transformations

Types of measures for forest-based bioeconomy transformations and related agents of change	Description	Examples of reviewed documents aligning with each type
Transformation through political support/restrictions	Supportive measures: - Investments in R&D, innovation, and upscaling to support the marketability of bio-based products - Public support for the wood construction sector, low-carbon public procurement policies, and raised taxes on fossils to enhance the competitiveness of bio-based products Restrictive measures: - Stronger legislation - Clear multilevel policy framework Key agents of change include industries, policymakers, and research institutes/universities	Borgström (2018), Cavicchi et al. (2017), Hagman et al. (2018), Husgafvel et al. (2018), Hurmekoski et al. (2018), Jarre et al. (2020), Johansson (2018), Kasatovaa et al. (2016), Lazarevic et al. (2020), Myking et al. (2017), Pannicke et al. (2015), Temmes and Peck (2020)
Transformation through inclusion, collaboration, and transparency	- Broad stakeholder participation to achieve inclusive, legitimate, transparent, and/or efficient bioeconomy transformations Strengthening cross-sectoral collaboration (as also suggested in the EU bioeconomy strategy) Key agents of change include a broad group of stakeholders such as entrepreneurs, farmers/forest owners, ENGOs, and citizens	Ahlqvist and Sirviö (2019), Asada and Stern (2018), Bennich et al. (2018), Giurca (2019), Giurca and Metz (2018), Giurca adn Späth (2018), Grundel and Dahlström (2016), Johansson (2018), Kröger and Raitio (2016), Näyhä (2019), Takala et al. (2019), Temmes and Peck (2020)
Transformation through information	- Information, primarily in terms of quantifiable indicators and targets - Environmental monitoring - Monitoring of bioeconomy development - Sustainability indicators - Corporate reporting Key agents of change include public and private actors, such as policymakers and business organizations	Budzinski et al. (2017), D’Amato et al. (2019), Husgafvel et al. (2018), Karvonen et al. (2017), Siebert et al. (2018), Sommerhuber et al. (2017)

#### Transformation through political support

Calls for (more) political backing is a recurrent measure presented as vital to bioeconomy transformations. Typical measures involve investments and institutional support for research and development (R&D) in the area of bioeconomy innovations, products, processes, and services in sectors ranging from forest-based biorefineries to forest management and product innovation (Lehtonen and Okkonen 2013; Hagemann et al. 2016; Jernström et al. 2017; Lilja and Moen 2017; Myking et al. 2017). Other advocated measures involve, e.g. increased taxes on fossil-based products/fuels (Pannicke et al. 2015) or supporting bio-based products through public procurement policies (Lazarevic et al. 2020), which aim at strengthening the entrance and competitiveness of bio-based technologies, products, and fuels on the market. This is especially present in papers that focus on wood construction or other bio-based products where political support for a bio-based market is called for (Hurmekoski et al. 2018; Toppinen et al. 2018). State support is primarily aimed at sharing costs and helping the private sector overcome market hurdles associated with substitution rather than at steering the bioeconomy transformation towards a certain desired end through, e.g. long-term and democratic planning at various administrative levels.

Although these studies attribute agency to the state, its role is limited to being a partner and facilitator. Despite its regulating power, not acknowledged as a ‘governor’ potentially imposing restricting legislations. Policymakers are primarily portrayed as facilitators that can provide companies/private sector/forest industries with beneficial conditions and remove barriers to the bioeconomy transformation through research funding and economic investments that stimulate innovation and upscaling. The strong emphasis on R&D entails that universities, research institutions, and researchers as a group are attributed a central role in the transition. Research called for in these publications generally involves the specific disciplines in which the publications are located (Lovric et al. 2020) (as is also the case with this article). Certain scholars call for more support for interdisciplinary research to foster innovation and/or as a way to address social and natural systems alike (Bennich et al. 2018; Johansson 2018).

Although politics and policymakers are imagined as central to bioeconomy transformations, primarily because they decrease uncertainties for market actors that explicitly or implicitly encompass the forest industries (e.g. wood construction, particle board industry, forest biorefinery clusters), few details are provided about who these policymakers are (be they elected representatives or bureaucrats), what public organizations they represent (e.g. ministries, state agencies, municipalities), and what concrete actions they should take at what administrative levels. Still, there are a few exceptions. Some studies do refer to specific ministries (e.g. ministries relate to forestry and agriculture) or mention advocacy coalitions between producers, consumers, politicians, and voters (Pannicke et al. 2015; Kröger and Raitio 2016).

Although less present, there are also alternative imaginaries of what policy support may imply. Cavicchi et al. (2017) argue that the dominance of industrial and national interests may jeopardize the sustainability of bio-based industries, exacerbate conflicts, and lead to a land-acquisition rush with unforeseen local environmental effects. As a result, Cavicchi and colleagues argue (ibid.), national governments need to develop clear policy frameworks (including, e.g. key objectives, targets, short- and long-term goals), whereas regional and local public authorities (municipalities) need to develop a varied and locally adapted range of initiatives. Similarly, Borgström (2018) argues for strengthening forest regulation to better manage land-use conflicts and secure ecological and social sustainability in bioeconomy transformations, including increased integration between forest law and other fields of law and policy important for the bioeconomy, such as climate and energy law, pollution control, and nature conservation.

#### Transformation through collaboration, inclusion, and transparency

Other measures represented as central for achieving sustainable forest-based bioeconomy transformations include collaboration, stakeholder inclusion/participation, and transparency. Studies that empirically focus on the private sector typically call for expansion of sector networks and enhanced cross-sector cooperation as ways to generate a shared identity of forest-based bioeconomy networks. This includes cross-sectoral collaboration between the forest-based sector and other industries to ensure resource-efficiency and foster innovations (Giurca and Späth 2017; Giurca 2020). Other articles identify and address problems in forest-based bioeconomy governance, including merits and pitfalls of collaborative governance and emphasize the importance of balancing competing forest values. Many of these studies see the forest-based bioeconomy as a potential pathway towards sustainability (see Sect. 4.2.2) and call for enhanced public participation and transparency combined with clear policy goals and/or strong forest regulation and policy coordination as important measures for achieving a sustainable and legitimate bioeconomy transformation (Pannicke et al. 2015; Borgström 2018; Hurmekoski et al. 2018; Johansson 2018). Participation and enhanced inclusiveness are not only ways to ensure democratic decision-making but also means to increase efficiency by reducing the risk of unforeseen consequences/effects of the strategies and measurements taken (Giurca and Metz 2018; Mustalahti 2018).

Whereas ‘stakeholders’ and ‘consumers’ are repeatedly referred to, and consideration of the values of the ‘broader public’ is encouraged without further specification, broad participation is generally seen as a prerequisite for a successful bioeconomy transformation. When specified, concerned stakeholders particularly include those outside of traditional forest-based industries such as citizens, consumers, NGOs, and local authorities (Grundel and Dahlström 2016; Korhonen et al. 2018a, b). Broad participation is also seen as a means to create a shared understanding of the bioeconomy (Näyhä 2019), to redefine and democratize the bioeconomy (Ahlqvist and Sirviö 2019), and to challenge established ways of thinking and doing and open up for alternative imaginaries (Takala et al. 2019).

#### Transformation through information

Information is a recurring instrument presented as vital to the monitoring, assessment, and achievement of sustainable bioeconomy transformations (Budzinski et al. 2017; Karvonen et al. 2017). Information in this regard includes corporate reporting that demonstrates sustainability performance at the company level (D'Amato et al. 2019) and the development of various criteria and indicators aimed at monitoring and/or assessing the sustainability of forest biomass extraction and bio-based products and fuels. Certain articles focus particularly on environmental impacts (May et al. 2017), others on social indicators (Siebert et al. 2018), and others on both environmental and social impacts (Sommerhuber et al. 2017).

In general, quantifiable indicators for the forest-based bioeconomy are portrayed as central instruments for assessing the sustainability performance of private and public actors, and as a means to guide consumers, producers, and markets in a sustainable direction. Another example is offered by studies investigating multistorey wood construction, which call for more education within the construction sector where architects and builders should be informed of the ‘whys’ and ‘hows’ of designing and building with wood. Suggestions include the standardization of skills and knowledge or by establishing learning routines (Toppinen et al. 2018).

## Discussion

Our analysis illustrates how social science research collectively co-produce rather homogenous imaginaries of forest-based bioeconomy transformations. Despite some diversity in the reviewed documents, the dominant way of seeing desired ends is through the lens of existing bioeconomy policies (particularly the EU bioeconomy strategy), which involves fossil independence, economic growth, and global competitiveness. From a co-productionist perspective, the replication of bioeconomy policies is problematic. As shown by Ramcilovik-Suominen and Pülzl (2016), the EU Bioeconomy imaginary entails a vision to maintain and increase the flow of goods and services along with current consumption levels and competitiveness. Inevitably, the research replicating these imaginaries tends to represent sustainability strictly in resource-efficiency terms and tends to take the sustainability of forest-based bioeconomy transformation for granted. Sustainability is thus often reduced to the use of renewable bio-based products and long-term sustained yields of forest biomass. Meanwhile, little consideration is given to social injustices and destructive local environmental effects (c.f. Pavone and Goven 2017, p. 5). However, considering that the recently revised EU Bioeconomy strategy reflects a more holistic approach to sustainability (European Commission 2018), future research replicating the EU bioeconomy agenda is likely to be more diverse and potentially more transformative.

The dominant imaginary of why forest-based bioeconomy transformations are necessary (for achieving a competitive fossil-free economy) is further reflected in the visions of what has to be changed. Accordingly, the technological and social objects of change are intimately related to forest industry renewal, including development and marketability of new technologies, materials, production processes, forest management practices, along with the increased use and cascading of forest biomass (Näyhä et al. 2015; Hagman et al. 2018; Hurmekoski et al. 2018; Hildebrandt et al. 2019). Social objects of change further include the establishment of new industrial collaboration networks among actors within and across bioeconomy sectors to foster innovation clusters (often on regional scales) (Giurca and Metz 2018; Korhonen et al. 2018a, b). Imaginaries of the forest-based bioeconomy transformation as imbued with changes in forest industrial production patterns (and consumption patterns at times) resemble a ‘Rubik’s cube approach’, whereby the bioeconomy is seen as a system where primarily the forest industries need to constantly move the parts seamlessly and more efficiently, always seeking perfect alignment to make the bioeconomy happen. If thinking of sustainable bioeconomy transformations as requiring fundamental shifts in human–environmental interactions, social inclusion, and linking local place-based needs with global approaches (c.f. Mancebo and Sachs 2015; Hölscher et al. 2018), research primarily aiming for more efficient forest industry production patterns offer few avenues for transformative action. This approach rather detaches the forest-based bioeconomy transformation from forest ecosystems and local realities, as they take a backseat in the race for global competitiveness and fossil independence (c.f. Pavone and Goven 2017). Furthermore, consumption, limits to growth or adverse effects on forest socio-ecological systems are rarely addressed, instead a vision of ‘more of everything’ or a ‘win–win’ solution prevails (Lindhal et al. 2017; Vivien et al. 2019).

Lastly, regarding how and by whom the bioeconomy is to be set in motion, the suggested measures typically involve soft and voluntary modes of governing, such as public funding investments in R&D, private–public collaboration (between forest industries, academia, and policymakers), broad stakeholder participation to create legitimacy for bioeconomy-related decisions and to avoid unforeseen effects, and information (e.g. environmental monitoring, corporate reporting, sustainability indicators) through which the sustainability performance of public and private organizations may be assessed and communicated to consumers and citizens. The role of the state is primarily to facilitate forest industry renewal, e.g. by providing substantial funding for research to relevant research institutes and universities for developing new processes, products, and upscale technologies. Although certain studies suggest restricting regulation (Borgström 2018) and a stronger role of national, regional, and local public authorities (Cavicchi et al. 2017), the advocated measures have a clear supportive purpose (Pannicke et al. 2015). This dominant imaginary of how and by whom the forest-based bioeconomy transformation should be governed favours market-based solutions and private–public partnerships (between the state, academia, and industry). It particularly illustrates the increasingly intimate collaboration between the life sciences and the state, prominently in the European Union (Jasanoff 2005). By nurturing and naturalizing this close collaboration between state-academy-industry in our research, which may be seen as a form of ‘forest-bio corporatism’ (c.f. Kröger and Raitio 2016), we also risk marginalizing a range of voices, places, and ecological functions that support the forest-based bioeconomy. This is a risk that has also been observed by several studies, primarily those that have a more problematizing and critical approach to forest-based bioeconomy transformations (c.f. Pülzl et al. 2014; Mustalahti 2018; Ahlqvist and Sirviö 2019; Takala et al. 2019). The reviewed studies predominantly replicate bioeconomy transformations as sustainable routes to fossil independence, and privilege supportive rather than restricting modes of governing. Therefore, little attention is paid to existing inequalities and how they may be addressed. Considering that rising levels of inequality may imply that those less affluent or influential may come to view bioeconomy transformations as elitist projects, it is important that social scientists pay additional attention to current and potential inequalities embedded in bioeconomy-related projects.

## Conclusions

Based on the review findings, the social science research on the forest-based bioeconomy replicates the desired goals, means and actors of change presented in existing policies, and particularly the EU bioeconomy strategy. This entails fossil independence, economic growth, and global competitiveness. How can social scientists working in the context of the bioeconomy move from the status quo in a constructive way? What research strategies can support social scientists in adopting different bioeconomy imaginaries in their work?

Despite the increasing number of publications, social-scientific research has had so far little impact on how existing bioeconomy policies and sectors are actually being shaped and transformed. As opposed to scholars in the natural sciences, social scientists have been rather wary in suggesting clear pathways to action. This is partly rooted in the complexity of the bioeconomy in the making, and in that social science research often bring light to win–lose relationships that require politically uncomfortable measures rather than technical win–win solutions. But it may also be a result of the different ontologies and schools of thought through which the bioeconomy is addressed, which make a clearer, more unified social-scientific research strategy on the bioeconomy difficult to achieve. Below we suggest some potential research strategies that may help overcome this conundrum.

One important research strategy that can provide nuances to the dominant policy rationale is to expand on research topics currently associated with forest-based bioeconomy transformation, such as the generation and distribution of socio-economic value and environmental costs (e.g. across global, national, and regional scales), distribution of public investments and future profit, and examination of what forest-related practices, products, and services are made relevant to public investments, R&D, and sustainability reporting. Although such research does not challenge the dominant policy rationale, it has implications for policymaking, as it potentially brings attention to certain aspects that are vital for well-informed and effective decision-making, including priorities and trade-offs between various interests and forest values.

An additional research strategy is to actively build on the research directions pointed out by the reviewed studies adopting a more problematizing and critical approach. This approach may also have different policy implications. The more descriptive and normative studies have implications for policy in the sense that they problematize established forest actors, institutions, and political processes, particularly from a democratic perspective. By doing so these studies, at least in theory, serve to make current policymaking more legitimate, democratic, and sustainable. On the other hand, studies drawing on critical theories generally challenge the sustainability of bioeconomy transformations from the start and avoid accepting established forest actors and institutions as the main (and only) point of departure (e.g. Ramcilovik-Suominen and Pülzl 2016; Kröger and Raitio 2016; Mustalahti 2018). By directing attention to overlooked forest functions, actors, scales, and places, new ways of thinking and imagining sustainable futures may be mapped out, potentially helping to re-envision and recalibrate our collective imaginaries of the forest-based bioeconomy. This includes how these imagined futures ought to be pursued and implemented here and now.

It is also important to seek interaction with other social science disciplines. We limited this review to a few social science disciplines previously presented as particularly relevant to forest-based bioeconomy research (Kleinschmit et al. 2014). By including a wider scope of social science disciplines, more diverse forest-based bioeconomy imaginaries than those identified in this review are likely to emerge. Such interdisciplinary engagement may help cultivate ways of thinking differently about forest-based bioeconomy products and services currently deemed important by policymakers and corporate actors. Different perspectives may allow research recommendations to move beyond the development of the right institutions, markets, and metrics. Possible disciplines include political ecology, a discipline engaged in the use and control of natural resources along with environmental change and its representations (Goldman and Turner 2011) or human geography, which may help direct attention to the scales and places of the bioeconomy and its implications for, e.g. equity and justice (Ahlqvist and Sirviö 2019). This is particularly relevant, as social science forest-based bioeconomy research is so far Euro-centric and particularly focused on Northern Europe (see Fig. 2). This also appears to be reflected in the distribution of bioeconomy-related research funding (Lovrić et al. 2020). Meanwhile, various regions of the world are engaging in bioeconomy policies creating resource interdependencies, geopolitical interests, asymmetric power relations, and winners and losers, which all need further scrutiny (Kröger 2016). Turning to more critical and interpretive social sciences may help in revisiting cultural and social assumptions that inform how researchers collectively make sense of sustainable development, of forests and human–forest relations, and of bioeconomy transformations (Lövbrand et al. 2015). Such a broadened social science research agenda on the forest-based bioeconomy highlights the fundamental political conflicts and choices imbued in bioeconomy transformations, which ‘forest-bio corporatism’ (Kröger and Raitio 2016) tends to conceal. As a result, more transformative political bioeconomy endeavours that are better equipped to make visible and handle the ecological, cultural, and ethical consequences of various policy choices.

Lastly, our intention with the review was not to evaluate the scientific contributions of individual scholars or of various social-scientific disciplines. Our hope is rather that the findings of this review will inspire critical reflexivity and jumpstart a discussion around the normative underpinnings of forest-based bioeconomy research, the research perspectives taken or neglected, and the collective shaping of the bioeconomy as a more or less transformative sustainability project. Although this is a challenging and uncomfortable task, irrespective of academic affiliation, such reflections and discussions are more relevant than ever in light of the urgency for swift policy action against the climate and biodiversity crises.

## Electronic supplementary material

Below is the link to the electronic supplementary material.

Supplementary file 1 (PDF 592 kb)

## Footnotes

1 Strong sustainability typically considers ecological viability and resilience as prerequisites for economic growth and human well-being.

2 References to the bioeconomy or bio-based economy from medical journals that included stem cell or blood research were not included in the review.
