# Correction to: Bioeconomy imaginaries: A review of forest-related social science literature

**DOI:** 10.1007/s13280-022-01713-3

**Published:** 2022-02-23

**Authors:** Sara Holmgren, Dalia D’Amato, Alexandru Giurca

**Affiliations:** 1 Department of Urban and Rural Development, Swedish University of Agricultural Sciences, Ulls väg 27, 750 07 Uppsala, Sweden; 2 Helsinki Institute of Sustainability Science, Department of Forest Sciences, Faculty of Agriculture and Forestry, University of Helsinki, Latokartanonkaari 7, P.O. Box 27, 00014 Helsinki, Finland; 3 Chair of Forest and Environmental Policy, Faculty of Environment and Natural Resources, University of Freiburg, Tennenbacherstr. 4, 79106 Freiburg, Germany

## Correction to: Ambio (2020) 49:1860–1877 10.1007/s13280-020-01398-6

In the original publication, the grant number 2016-00857 was missed mistakenly in the acknowledgements section. Below is the correct acknowledgements of the article.

**Acknowledgements** Open access funding provided by University of Helsinki including Helsinki University Central Hospital. This study was funded through the Swedish Research Council for Sustainable Development (FORMAS) (Reg. No. 942-2016-30, 2016-00857), Academy of Finland (projects OPES, funding decision no. 315912), and European Forest Institute (EFI) through its EFI Network Fund initiative. The publication is part of the PerForm project, a collaboration network of social scientists from across Europe, investigating societal perceptions of the forest-based bioeconomy.

The original article has been corrected.

