# Supplementary material for: Bioeconomy imaginaries: A review of forest-related social science literature
Source: Ambio. 2020 Oct 9;49(12):1860–77. doi: 10.1007/s13280-020-01398-6 (PMC7568747; doi:10.1007/s13280-020-01398-6)
Supplement: Supplementary file 1 — (PDF 592 kb) [file 13280_2020_1398_MOESM1_ESM.pdf]

Ambio

Electronic Supplementary Material
This supplementary material has not been peer reviewed.

Title: **Bioeconomy Imaginaries: A review of forest-related social science literature**
Authors: Sara Holmgren, Dalia D'Amato, Alexandru Giurca

Appendix 1 Articles reviewed

1. Ahlqvist, T., and H. Sirviö. 2019. Contradictions of Spatial Governance: Bioeconomy and the Management of State Space in Finland. *Antipode* 51. John Wiley & Sons, Ltd (10.1111): 395–418. doi:10.1111/anti.12498.
2. Asada, R., and T. Stern. 2018. Competitive Bioeconomy? Comparing Bio-based and Non-bio-based Primary Sectors of the World. *Ecological Economics* 149. Elsevier: 120–128. doi:10.1016/J.ECOLECON.2018.03.014.
3. Bais-Moleman, A. L., R. Sikkema, M. Vis, P. Reumerman, M. C. Theurl, and K.-H. Erb. 2018. Assessing wood use efficiency and greenhouse gas emissions of wood product cascading in the European Union. *Journal of Cleaner Production* 172. Elsevier: 3942–3954. doi:10.1016/J.JCLEPRO.2017.04.153.
4. Bennich, T., S. Belyazid, B. Kopainsky, and A. Diemer. 2018. The Bio-Based Economy: Dynamics Governing Transition Pathways in the Swedish Forestry Sector. *Sustainability* 10. Multidisciplinary Digital Publishing Institute: 976. doi:10.3390/su10040976.
5. Birch, K., L. Levidow, and T. Papaioannou. 2010. Sustainable Capital? The Neoliberalization of Nature and Knowledge in the European "Knowledge-based Bio-economy." *Sustainability* 2. Molecular Diversity Preservation International: 2898–2918. doi:10.3390/su2092898.
6. Blair, M. J., L. Cabral, and W. E. Mabee. 2017. Biorefinery strategies: exploring approaches to developing forest-based biorefinery activities in British Columbia and Ontario, Canada. *Technology Analysis & Strategic Management* 29. Routledge: 528–541. doi:10.1080/09537325.2016.1211266.
7. Borgström, S. 2018. Reviewing natural resources law in the light of bioeconomy: Finnish forest regulations as a case study. *Forest Policy and Economics* 88. Elsevier: 11–23. doi:10.1016/J.FORPOL.2017.10.012.
8. Budzinski, M., A. Bezama, and D. Thrän. 2017. Monitoring the progress towards bioeconomy using multi-regional input-output analysis: The example of wood use in Germany. *Journal of Cleaner Production* 161. Elsevier: 1–11. doi:10.1016/J.JCLEPRO.2017.05.090.
9. Cavicchi, B., S. Palmieri, and M. Odaldi. 2017. The Influence of Local Governance: Effects on the Sustainability of Bioenergy Innovation. *Sustainability* 9. Multidisciplinary Digital Publishing Institute: 406. doi:10.3390/su9030406.
10. D'Amato, D., J. Korhonen, and A. Toppinen. 2019. Circular, Green, and Bio Economy: How Do Companies in Land-Use Intensive Sectors Align with Sustainability Concepts? *Ecological*

Economics 158. Elsevier: 116–133. doi:10.1016/J.ECOLECON.2018.12.026.

11. Eyvindson, K., A. Repo, and M. Mönkkönen. 2018. Mitigating forest biodiversity and ecosystem service losses in the era of bio-based economy. *Forest Policy and Economics* 92. Elsevier: 119–127. doi:10.1016/J.FORPOL.2018.04.009.
12. Giurca, A. 2020. Unpacking the network discourse: Actors and storylines in Germany's wood-based bioeconomy. *Forest Policy and Economics* 110. Elsevier: 101754. doi:10.1016/J.FORPOL.2018.05.009.
13. Giurca, A., and T. Metz. 2017. A social network analysis of Germany's wood-based bioeconomy: Social capital and shared beliefs. *Environmental Innovation and Societal Transitions*. Elsevier: 1–14. doi:10.1016/j.eist.2017.09.001.
14. Giurca, A., and P. Späth. 2017. A Forest-based Bioeconomy for Germany? Strengths, Weaknesses and Policy Options for Lignocellulosic Biorefineries. *Journal of Cleaner Production* 153: 51–62. doi:10.1016/j.jclepro.2017.03.156.
15. Grundel, I., and M. Dahlström. 2016. A Quadruple and Quintuple Helix Approach to Regional Innovation Systems in the Transformation to a Forestry-Based Bioeconomy. *Journal of the Knowledge Economy* 7. Springer: 963–983. doi:10.1007/s13132-016-0411-7.
16. Hagemann, N., E. Gawel, A. Purkus, N. Pannicke, and J. Hauck. 2016. Possible Futures towards a Wood-Based Bioeconomy: A Scenario Analysis for Germany. *Sustainability* 8: 98. doi:10.3390/su8010098.
17. Hagman, L., A. Blumenthal, M. Eklund, and N. Svensson. 2018. The role of biogas solutions in sustainable biorefineries. *Journal of Cleaner Production* 172. Elsevier: 3982–3989. doi:10.1016/J.JCLEPRO.2017.03.180.
18. Hildebrandt, J., S. O'Keeffe, A. Bezama, and D. Thrän. 2019. Revealing the Environmental Advantages of Industrial Symbiosis in Wood-Based Bioeconomy Networks: An Assessment From a Life Cycle Perspective. *Journal of Industrial Ecology* 23. John Wiley & Sons, Ltd (10.1111): 808–822. doi:10.1111/jiec.12818.
19. Hurmekoski, E., R. Jonsson, J. Korhonen, J. Jänis, M. Mäkinen, P. Leskinen, and L. Hetemäki. 2018a. Diversification of the forest industries: role of new wood-based products. *Canadian Journal of Forest Research* 48. NRC Research Press: 1417–1432. doi:10.1139/cjfr-2018-0116.
20. Hurmekoski, E., J. Pykäläinen, and L. Hetemäki. 2018b. Long-term targets for green building: Explorative Delphi backcasting study on wood-frame multi-story construction in Finland. *Journal of Cleaner Production* 172. Elsevier: 3644–3654. doi:10.1016/J.JCLEPRO.2017.08.031.
21. Hurmekoski, E., M. Lovrić, N. Lovrić, L. Hetemäki, and G. Winkel. 2019. Frontiers of the forest-based bioeconomy – A European Delphi study. *Forest Policy and Economics* 102. Elsevier: 86–99. doi:10.1016/J.FORPOL.2019.03.008.

22. Husgafvel, R., L. Linkosalmi, M. Hughes, J. Kanerva, and O. Dahl. 2018. Forest sector circular economy development in Finland: A regional study on sustainability driven competitive advantage and an assessment of the potential for cascading recovered solid wood. *Journal of Cleaner Production* 181. Elsevier: 483–497. doi:10.1016/J.JCLEPRO.2017.12.176.
23. Jarre, M., A. Petit-Boix, C. Priefer, R. Meyer, and S. Leipold. 2020. Transforming the bio-based sector towards a circular economy - What can we learn from wood cascading? *Forest Policy and Economics* 110. Elsevier: 101872. doi:10.1016/J.FORPOL.2019.01.017.
24. Jernström, E., V. Karvonen, T. Kässi, A. Kraslawski, and J. Hallikas. 2017. The main factors affecting the entry of SMEs into bio-based industry. *Journal of Cleaner Production* 141: 1–10. doi:https://doi.org/10.1016/j.jclepro.2016.08.165.
25. Johansson, J. 2016. Participation and deliberation in Swedish forest governance: The process of initiating a National Forest Program. *Forest Policy and Economics* 70. Elsevier: 137–146. doi:10.1016/J.FORPOL.2016.06.001.
26. Johansson, J. 2018. Collaborative governance for sustainable forestry in the emerging bio-based economy in Europe. *Current Opinion in Environmental Sustainability* 32. Elsevier: 9–16. doi:10.1016/J.COSUST.2018.01.009.
27. Karvonen, J., P. Halder, J. Kangas, and P. Leskinen. 2017. Indicators and tools for assessing sustainability impacts of the forest bioeconomy. *Forest Ecosystems* 4. SpringerOpen: 2. doi:10.1186/s40663-017-0089-8.
28. Kasatovaa, A., V. I. Vagizovaa, and A. M. Tufetulova. 2016. BIOECONOMY'S POTENTIAL FOR DEVELOPMENT AND COMMERCIALIZATION OPPORTUNITIES FOR BIOSPHERE PROJECTS IN RUSSIA. *Electronic Business Journal* 15: 363.
29. Kleinschmit, D., B. J. Arts, A. Giurca, I. Mustalahti, A. Sergent, and H. Pulzl. 2017. Environmental concerns in political bioeconomy discourses. *International Forestry Review* 19: 1–15.
30. Korhonen, J., E. Hurmekoski, E. Hansen, and A. Toppinen. 2018a. Firm-level competitiveness in the forest industries: Review and research implications in the context of bioeconomy strategies. *Canadian Journal of Forest Research* 48: 141–152. doi:10.1139/cjfr-2017-0219.
31. Korhonen, J, A. Giurca, M. Brockhaus, A. Toppinen, Jaana Korhonen, A. Giurca, M. Brockhaus, and A. Toppinen. 2018b. Actors and Politics in Finland's Forest-Based Bioeconomy Network. *Sustainability* 10. Multidisciplinary Digital Publishing Institute: 3785. doi:10.3390/su10103785.
32. Kröger, M. 2016. The political economy of 'flex trees': a preliminary analysis. *The Journal of Peasant Studies* 43. Routledge: 886–909. doi:10.1080/03066150.2016.1140646.
33. Kröger, M., and K. Raitio. 2016. Forest Policy and Economics Finnish forest policy in the era of

bioeconomy : A pathway to sustainability ? *Forest Policy and Economics*. Elsevier B.V. doi:10.1016/j.forpol.2016.12.003.

34. Lazarevic, D., P. Kautto, and R. Antikainen. 2020. Finland's wood-frame multi-storey construction innovation system: Analysing motors of creative destruction. *Forest Policy and Economics* 110. Elsevier: 101861. doi:10.1016/J.FORPOL.2019.01.006.
35. Lehtonen, O., and L. Okkonen. 2013. Regional socio-economic impacts of decentralised bioeconomy: a case of Suutela wooden village, Finland. *Environment, Development and Sustainability* 15. Springer Netherlands: 245–256. doi:10.1007/s10668-012-9372-6.
36. Lilja, K., and E. Moen. 2017. Orchestrating a new industrial field. The case of the Finnish wood-based bioeconomy. *International Journal of Business Environment* 9: 266. doi:10.1504/IJBE.2017.087975.
37. Lochhead, K., S. Ghafghazi, P. Havlik, N. Forsell, M. Obersteiner, G. Bull, and W. Mabee. 2016. Price trends and volatility scenarios for designing forest sector transformation. *Energy Economics* 57. North-Holland: 184–191. doi:10.1016/J.ENECO.2016.05.001.
38. Lovrić, M., N. Lovrić, and R. Mavsar. 2020. Mapping forest-based bioeconomy research in Europe. *Forest Policy and Economics* 110. Elsevier: 101874. doi:10.1016/J.FORPOL.2019.01.019.
39. Matthies, B. D., A. Vainio, and D. D'Amato. 2018. Not so biocentric – Environmental benefits and harm associated with the acceptance of forest management objectives by future environmental professionals. *Ecosystem Services* 29. Elsevier: 128–136. doi:10.1016/J.ECOSER.2017.12.003.
40. May, N., E. Guenther, and P. Haller. 2017. Environmental Indicators for the Evaluation of Wood Products in Consideration of Site-Dependent Aspects: A Review and Integrated Approach. *Sustainability* 9. Multidisciplinary Digital Publishing Institute: 1897. doi:10.3390/su9101897.
41. Mustalahti, I. 2017. The responsive bioeconomy: The need for inclusion of citizens and environmental capability in the forest based bioeconomy. *Journal of Cleaner Production*. Elsevier Ltd. doi:10.1016/j.jclepro.2017.06.132.
42. Myking, T., M. Walløe Tvedt, and B. Karlsson. 2017. Protection of forest genetic resources by intellectual property rights – exploring possibilities and conceivable conflicts. *Scandinavian Journal of Forest Research* 32. Taylor & Francis: 598–606. doi:10.1080/02827581.2017.1293151.
43. Näyhä, A. 2019. Transition in the Finnish forest-based sector: Company perspectives on the bioeconomy, circular economy and sustainability. *Journal of Cleaner Production* 209. Elsevier: 1294–1306. doi:10.1016/J.JCLEPRO.2018.10.260.

44. Näyhä, A., P. Pelli, and L. Hetemäki. 2015. Services in the forest-based sector – unexplored futures. Edited by Ms. Deborah Cox, Dr. Lawrence Green, and Dr. Krzysztof Borodako. *Foresight* 17. Emerald Group Publishing Limited: 378–398. doi:10.1108/FS-08-2013-0034.
45. Pannicke, N., E. Gawel, N. Hagemann, A. Purkus, and S. Strunz. 2015. The Political Economy of Fostering a Wood-based Bioeconomy in Germany. *German Journal Agricultural Economics* 64: 224–243.
46. Pätäri, S., H. Arminen, L. Albareda, K. Puumalainen, and A. Toppinen. 2017. Student values and perceptions of corporate social responsibility in the forest industry on the road to a bioeconomy. *Forest Policy and Economics* 85. Elsevier: 201–215. doi:10.1016/J.FORPOL.2017.10.009.
47. Pelli, P., A. Haapala, and J. Pykäläinen. 2017. Services in the forest-based bioeconomy – analysis of European strategies. *Scandinavian Journal of Forest Research* 32. Taylor & Francis: 559–567. doi:10.1080/02827581.2017.1288826.
48. Pelse, M., D. Ziedina, L. Aleksejeva, and M. Bitmane. 2018. COOPERATION AS A SUSTAINABLE FACTOR INFLUENCING INNOVATION IN REGIONAL DEVELOPMENT: THE CASE OF THE BIOECONOMY IN LATVIA. *Journal of Security and Sustainability Issues* 7. Journal of Security and Sustainability Issues: 581–590. doi:10.9770/jssi.2018.7.3(17).
49. Purkus, A., N. Hagemann, N. Bedtke, and E. Gawel. 2018. Towards a sustainable innovation system for the German wood-based bioeconomy: Implications for policy design. *Journal of Cleaner Production*. doi:10.1016/j.jclepro.2017.04.146.
50. Siebert, A., S. O'Keeffe, A. Bezama, W. Zeug, and D. Thrän. 2018a. How not to compare apples and oranges: Generate context-specific performance reference points for a social life cycle assessment model. *Journal of Cleaner Production* 198. Elsevier: 587–600. doi:10.1016/J.JCLEPRO.2018.06.298.
51. Siebert, A., A. Bezama, S. O'Keeffe, and D. Thrän. 2018b. Social life cycle assessment indices and indicators to monitor the social implications of wood-based products. *Journal of Cleaner Production* 172. Elsevier: 4074–4084. doi:10.1016/J.JCLEPRO.2017.02.146.
52. Sikkema, R., J. F. Dallemand, C. T. Matos, M. van der Velde, and J. San-Miguel-Ayanz. 2016. How can the ambitious goals for the EU's future bioeconomy be supported by sustainable and efficient wood sourcing practices? *Scandinavian Journal of Forest Research*. Taylor & Francis: 1–8. doi:10.1080/02827581.2016.1240228.
53. Sommerhuber, P. F., J. L. Wenker, S. Rüter, and A. Krause. 2017. Life cycle assessment of wood-plastic composites: Analysing alternative materials and identifying an environmental sound end-of-life option. *Resources, Conservation and Recycling* 117. Elsevier: 235–248. doi:10.1016/J.RESCONREC.2016.10.012.

54. Takala, T., J. Tikkanen, A. Haapala, S. Pitkänen, P. Torssonen, R. Valkeavirta, and T. Pöykkö. 2019. Shaping the concept of bioeconomy in participatory projects – An example from the post-graduate education in Finland. *Journal of Cleaner Production* 221. Elsevier: 176–188. doi:10.1016/J.JCLEPRO.2019.02.007.
55. Temmes, A., and P. Peck. 2020. Do forest biorefineries fit with working principles of a circular bioeconomy? A case of Finnish and Swedish initiatives. *Forest Policy and Economics* 110. Elsevier: 101896. doi:10.1016/J.FORPOL.2019.03.013.
56. Toppinen, A., A. Röhr, S. Pätäri, K. Lähtinen, and R. Toivonen. 2018. The future of wooden multistory construction in the forest bioeconomy - A Delphi study from Finland and Sweden. *Journal of Forest Economics*. Elsevier GmbH.: 1–8. doi:10.1016/j.jfe.2017.05.001.
57. Toppinen, A., M. Sauru, S. Pätäri, K. Lähtinen, and A. Tuppura. 2019. Internal and external factors of competitiveness shaping the future of wooden multistory construction in Finland and Sweden. *Construction Management and Economics* 37. Routledge: 201–216. doi:10.1080/01446193.2018.1513162.
58. Watanabe, C., N. Naveed, and P. Neittaanmäki. 2018. Digital solutions transform the forest-based bioeconomy into a digital platform industry - A suggestion for a disruptive business model in the digital economy. *Technology in Society* 54. Pergamon: 168–188. doi:10.1016/J.TECHSOC.2018.05.002.
59. Watanabe, C., N. Naveed, and P. Neittaanmäki. 2019. Digitalized bioeconomy: Planned obsolescence-driven circular economy enabled by Co-Evolutionary coupling. *Technology in Society* 56. Pergamon: 8–30. doi:10.1016/J.TECHSOC.2018.09.002.
